# Combined endophthalmitis and orbital cellulitis in patients with corona virus disease (COVID-19)

**DOI:** 10.1186/s12348-021-00258-y

**Published:** 2021-09-15

**Authors:** Mohamed Farouk Sayed Othman Abdelkader, Ahmed Mohamed Kamal Elshafei, Mahmoud Mamdouh Nassar, Mostafa A. Abu Elela, Raafat Mohyeldeen Abdelrahman Abdallah

**Affiliations:** 1 Ophthalmology Department, Faculty of Medicine, Minia University, El-Minia, 61519 Egypt; 2 Department of Clinical pathology, Faculty of Medicine, Minia University, El-Minia, Egypt

**Keywords:** COVID-19, Orbital cellulitis, Endophthalmitis

## Abstract

**Purpose:**

To document the presentation of unilateral combined endophthalmitis and orbital cellulitis in patients with COVID-19 infection and study their prognosis.

**Patients and methods:**

This interventional case series study included 9 patients referred to the Ophthalmology Department, Minia University Hospital with unilateral combined endophthalmitis and orbital cellulitis between April 2020 and March 2021. In addition to the COVID-19 work-up, all patients were subjected to full ophthalmological evaluation and managed according to their ophthalmic and systemic disease.

**Results:**

The patients were 5 females and 4 males. They had clinical, laboratory and imaging findings that confirmed COVID-19 infection. All patients had unilateral endophthalmitis with orbital cellulitis and profound visual loss in the affected eye. Three patients died due to respiratory failure, while 6 patients recovered systemically. The survived patients developed atrophia bulbi in 4 patients and in 2 patients, the globe retained normal size but with complete visual loss.

**Conclusion:**

Combined endophthalmitis and orbital cellulitis can be one of the early presentations of patients with COVID-19 infection with poor visual prognosis.

**Trial registration:**

Clinical registration: clinicaltrials.gov identifier: NCT04456556.

## Introduction

Corona viruses represent a group of viruses that can cause respiratory infections ranging from common cold-like manifestations to more severe diseases such as Middle East Respiratory Syndrome (MERS) and Severe Acute Respiratory Syndrome (SARS). Severe acute respiratory syndrome coronavirus-2 (SARS-COV-2) is the most recently discovered corona virus [1]. It is a novel enveloped, positive single-stranded RNA virus that was originally linked to an outbreak in Wuhan, China [2]. Li Wenliang, a Chinese ophthalmologist, was the first to suspect the presence of Corona virus in his patients. Unfortunately, he later died from SARS-COV-2 infection transmitted from an asymptomatic glaucoma patient in his clinic. Ophthalmic manifestations of COVID-19 are uncommon, with most commonly reported is mild follicular conjunctivitis which is indistinguishable from other viral conjunctivitis [3]. Daruich et, al reported a 28 years old male patient with COVID-19 first presented with foreign body sensation and redness of his left eye with unilateral eyelid edema and moderate conjunctival hyperemia [4]. Turbin et al., reviewed 2 adolescents with COVID-19 presented with orbital cellulitis, sinusitis and intracranial abnormalities [5]. We managed 9 patients with COVID-19 with acute unilateral ocular pain, proptosis and visual loss secondary to combined endophthalmitis and orbital cellulitis.

## Patients and methods

This was an interventional case series study which included 9 patients referred to the Ophthalmology Department of Minia University Hospital (a tertiary ophthalmology center in Upper Egypt) with visual loss, ocular and orbital inflammation between April 2020 and March 2021. The study was approved by the local research ethical committee of the Faculty of Medicine, Minia University and was adherent to the tents of the Declaration of Helsinki. Informed written consents were obtained from all patients. These patients were not diagnosed to have Covid-19 infection before referral to our hospital with the presentation of ocular pain, proptosis, and defective vision and had not received any treatment in other health care facilities. Covid infection was proved only after ophthalmological presentation. Patients started to complain of ophthalmic manifestations 3–6 days before asking for medical advice. Once patients had presented to other healthcare providers, they were referred to our tertiary center in the same day. All patients were subjected to complete ophthalmological evaluation including detailed history, slit lamp examination, pupillary reflexes, ocular motility testing, and documentation of the visual acuity. ENT consultation was done to all patients to exclude the possibility of mucormycosis. Ocular ultrasonography and orbital computed tomography (CT) scan were done. All patients had fever on presentation and consequently, according to the regulations of the Egyptian Ministry of Health, systemic medical evaluation for COVID-19 infection was done for all patients; this included history taking for fever, respiratory symptoms (sore throat, cough, or dyspnea), loss of taste and smell, and gastrointestinal symptoms. Systemic medical examination was done including measurement of body temperature and oxygen saturation. Chest CT, and laboratory investigations including complete blood count (CBC) and polymerase chain reaction (PCR) of nasopharyngeal swabs for COVID-19. Also, PCR of conjunctival swabs for COVID-19 was performed. As the preliminary diagnosis was combined orbital cellulitis and endophthalmitis, intravenous broad-spectrum antibiotics covering gram-positive, gram-negative, and anaerobic bacteria were given to all patients, including a combination of IV vancomycin and ceftazidime with oral metronidazole. Topical antibiotics (moxifloxacin hydrochloride 0.5% eye drops), topical combination of dexamethasone and tobramycin, and cycloplegic (cyclopentolate 1%) eye drops were given to all patients. Vitreous tap with intravitreal injection of vancomycin (1 mg/0.1 ml) and ceftazidime (2.25/0.1 ml) was done and injection was repeated once after 48–72 h. Aqueous and vitreous samples were sent for microbiological testing. All patients were examined every day and received subconjunctival injection of antibiotics (vancomycin and ceftazidime) and dexamethasone. All patients were isolated and given the systemic medical regimen related to COVID-19 as recommended by the Egyptian Ministry of Health.

## Results

This study included 9 eyes of 9 patients (5 females and 4 males). The age of patients ranged between 12 to 75 years. All patients presented with acute unilateral painful visual loss since 3–7 days (mean 4.7 ± 1.32 days) and fever (38–39 C°). Four patients had dyspnea and dry cough. Chest CT scan findings of all patients were suggestive of COVID-19 infection with multiple patchy ground glass opacities scattered in both lung fields. All patients had laboratory findings of hypochromic microcytic anemia with hemoglobin ranged between 5.8–9.2 g/dl (mean 7.5 ± 1.2 mg/dl), increased total leucocytic count ranged between 12,700–22,300 (mean 19,266.7 ± 3437.3), and relative lymphocytopenia between 7 and 16% (mean 12.6 ± 2.9%) and positive PCR of nasopharyngeal swabs for COVID-19. Apart from one patient who had a history of renal failure on hemodialysis, there was no history of systemic debilitating diseases such as uncontrolled diabetes mellitus, malignancy, collagen diseases, or systemic steroid or immunosuppressant drug intake. One patient had a history of abortion 2 weeks earlier and one patient had a perforated corneal ulcer.

**Fig. 1:**
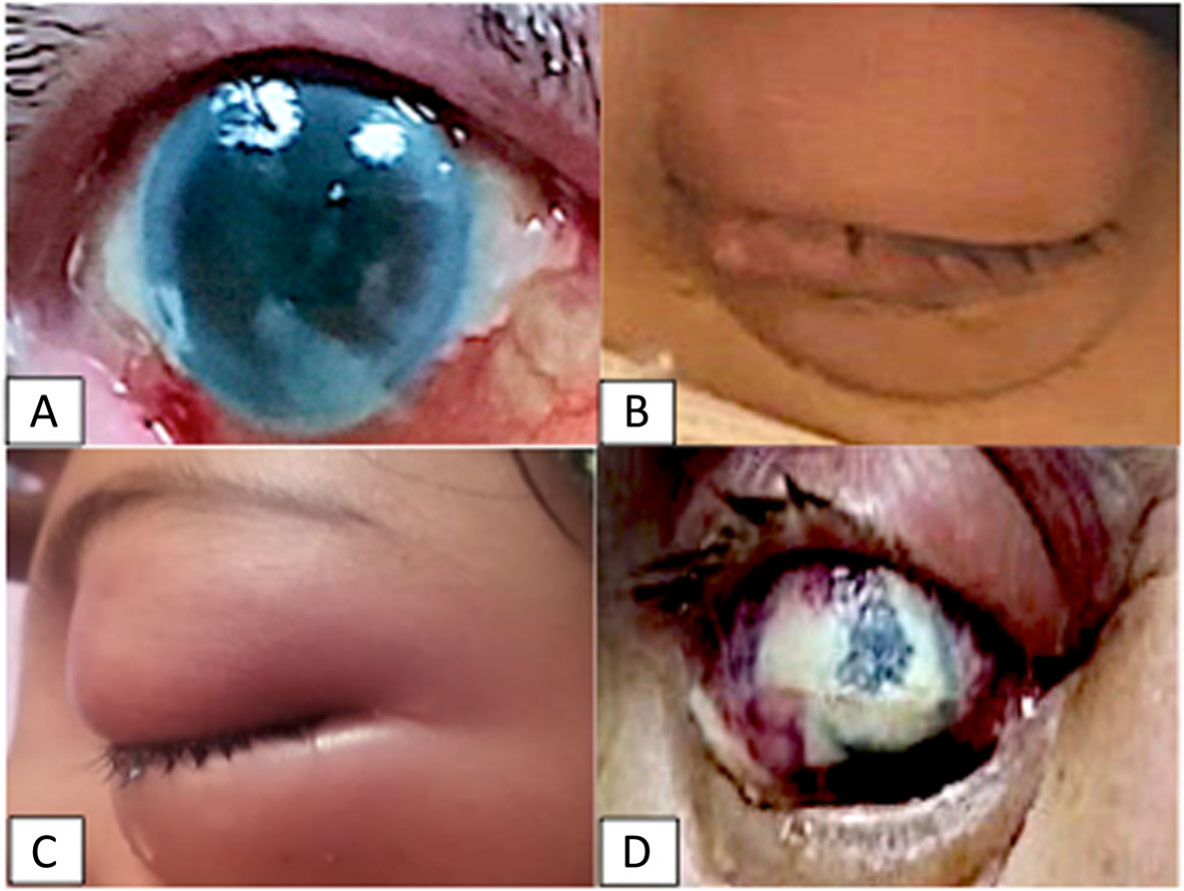
Clinical images of covid-19 patients with orbital cellulitis and endophthalmitis

**Fig. 2:**
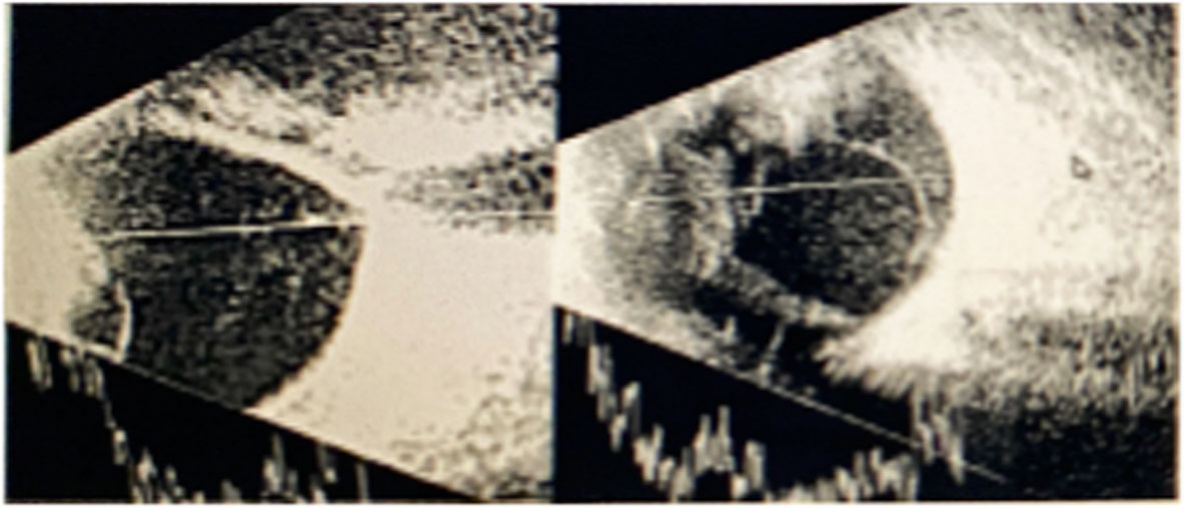
B-scan ocular ultrasonography shows medium to highly reflective floaters and membranous echoes with loculated opacities in the vitreous cavity more condensed posteriorly with choroidal thickening

**Fig. 3:**
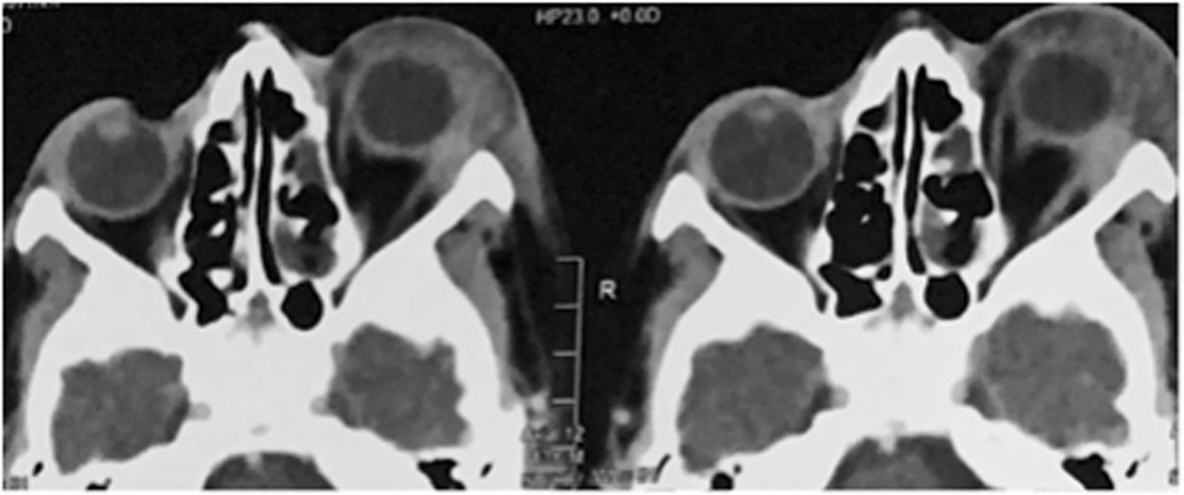
Axial orbital CT scan showing left orbital cellulitis with axial proptosis

On examination, all patients had edema and erythema of eye lids, severe conjunctival and ciliary injection, subconjunctival hemorrhage, and corneal edema and infiltration, in addition to dense inflammatory coagulum in the anterior chamber (Fig. 1). All patients had axial proptosis and limitation of the ocular motility. Visual acuity on presentation was no perception of light in 5 cases, perception of light with bad light projection in 2 cases, and hand motion with good projection in 2 patients. None of the patients had black eschars either in the periorbital area, face, nasal cavity, paranasal sinuses, or the hard palate which may give rise suspicion to mucormycosis. Ocular B-scan ultrasonography showed medium to highly reflective floaters and membranous echoes with loculated opacities in the vitreous cavity more condensed posteriorly with choroidal thickening and the retina was in place (Fig. 2). Orbital CT scan showed mild proptosis, haziness of orbital fat, in all cases. Mucoperiosteal thickening of the ethmoidal sinuses was observed in only one case (Fig. 3). The clinical data of the 9 cases were summarized in Table 1.

**Table 1:** Clinical data of the patients with combined endophthalmitis and orbital cellulitis

No.	Age/ Gender/eye	Duration of ocular symptoms	Visual acuity	Systemic signs	Systemic History	Conjunctival swab	Fate
1	60y/ Female /OD	6 days	NPL	Fever/cough/dyspnea +ve nasopharngeal PCR	Negative	Positive	death
2	55y/Female /OS	5 days	NPL	Fever/ cough/dyspnea/ +ve nasopharngeal PCR	Hypertension Renal failure	Negative	death
3	12y/Male /OS	3 days	HM	Fever/+ve nasopharngeal PCR	Negative	Negative	**N**PL
4	35y/Female /OS	5 days	NPL	Fever/+ve nasopharngeal PCR	Recent abortion	Negative	atrophia
5	65y/Male /OD	4 days	HM	Fever/+ve nasopharngeal PCR	Phaco+IOL 5 months ago	Negative	**N**PL
6	47y/Female /OD	3 days	PL	Fever/+ve nasopharngeal PCR	Negative	Negative	atrophia
7	75y/Female OS	4 days	NPL	Fever/ cough/dyspnea Cough/+ve nasopharngeal PCR	Perforated corneal ulcer	Positive	death
8	63/Male /OD	5	PL	Fever/+ve nasopharngeal PCR	Negative	Negative	atrophia
9	71/Male OS	6	NPL	Fever/ cough/dyspnea/+ve nasopharngeal PCR	Phaco+IOL 7y	Negative	atrophia

*HM* Vision is Hand motion, *NPL* Vision is no perception of light, *PL* Vision is perception of light, *PCR* Polymerase chain reaction, *Atrophia* Atrophy of eye globe

PCR of conjunctival swabs was positive in 2 patients (22%). Microbiological testing of aqueous and vitreous aspirates on aerobic and anerobic, and fungal media were negative. Three patients had progressive drop of oxygen saturation few days after hospitalization and were put on mechanical ventilator in the intensive care unit and they died. The other 6 patients were generally stable, and the orbital inflammation regressed with poor visual prognosis (atrophia bulbi in 4 eyes and preserved eyeball with complete visual loss in 2 patients).

## Discussion

COVID-19 is a very contagious disease that can progress to acute respiratory distress and can lead to death. There are few reports about different ophthalmic manifestations of this disease. In addition to the previous reports of follicular conjunctivitis in COVID-19 [3, 4], Chen et al., also reported a case of bilateral acute conjunctivitis in a 30-year-old patient with confirmed COVID-19. Conjunctivitis occurred 13 days after illness onset in the form of bilateral moderate conjunctival injection, watery discharge, inferior palpebral conjunctival follicles and tender palpable preauricular lymph nodes [6]. In patients with conjunctivitis, the virus may be present in tears. It could be detected in conjunctival swab samples using RT- PCR. As a result, the virus could be transmitted from the ocular surface to a new patient through contact with the ocular mucosa, tears, or fomites [7]. In the current study, positive PCR of conjunctival swab for COVID-19 was found in 2 cases. Turbin et al., reported two teenagers with COVID-19 presenting with orbital cellulitis, sinusitis, and intracranial abnormalities. The first case was an early adolescent patient presented with a history of progressive painful unilateral orbital swelling for 3 days. He had severe unilateral right upper and lower eyelid edema with mild erythema, non-hemorrhagic conjunctival chemosis, 3–4 mm proptosis, normal visual acuity and mild right afferent pupil defect. He had some limitation of ocular motility. Orbital CT showed right frontal, maxillary and anterior ethmoid sinusitis with subperiosteal fluid collection. The second case was an early adolescent patient with severe right upper and lower eyelid and periorbital edema, 3–4 mm of right eye proptosis, moderate limitation of the ocular motility, normal visual acuity and normal pupillary reaction. Also, paranasal sinusitis was present in CT [5]. A report from the American Academy of Ophthalmology stated that there is a possible link between devastating eye infection and COVID-19 and in just 2 months, three patients at New York’s Northwell Health hospital were diagnosed with keratitis that quickly led to endophthalmitis and profound vision loss. One patient’s eye had to be removed. All three patients tested positive for COVID-19. Two were outpatients and one was in the hospital at the time of keratitis diagnosis. One of the outpatients was later admitted to the hospital and died [8]. In the current study, one patient had perforated corneal ulcer with endophthalmitis and orbital cellulitis and died few days after hospitalization.

In this study, 9 patients were referred to Minia University Hospital with unusual presentation of acute simultaneous endophthalmitis and orbital cellulitis. All were proved to have COVID-19 infection by positive PCR of nasopharyngeal swabs. Minia University Hospital provides ophthalmic emergency care and tertiary service for a population of about 6 million people in upper Egypt. In a previous study, published in 2017 we reviewed 102 cases of orbital cellulitis treated in Minia University Hospital over a period of 6 years. In this relatively large number of patients, only 6 cases had orbital cellulitis combined with endophthalmitis secondary to extension from intraocular infection. Only 12.5% of cases had visual acuity of no perception of light while most cases had best corrected visual acuity better than 6/60 [9]. Generally, the combination of endophthalmitis and orbital cellulitis is uncommon; it either represents extension from intraocular infection through the sclera to cause panophthalmitis and orbital cellulitis, or conversely, neglected untreated cases of orbital cellulitis progressing to corneal abscess secondary to corneal exposure or due to extension of infection through the sclera or its foramina causing intraocular affection. This occurs in neglected untreated cases for relatively long period. However, in the current study this combination of endophthalmitis and orbital cellulitis presented simultaneously, early in the course of the disease, with severe visual loss and was the first presenting feature of the patients. Three possibilities could be postulated. Firstly, there may be generalized lowering of immunity, causing endogenous endophthalmitis and orbital cellulitis in patients with Covid-19. All patients had anemia and one patient had chronic renal failure. Paranasal sinusitis was present in only one case and was mild, suggesting the presence of a distant source of infection. Endogenous endophthalmitis (EE) is a rare disease and only accounts for approximately 2–8% of all cases of endophthalmitis [10]. Endogenous endophthalmitis results from metastatic spread of the organism from a primary site of infection in the setting of bacteremia or fungemia [11]. EE is often related to underlying systemic risk factors, including recent hospitalization, diabetes mellitus, urinary tract infection, immunosuppression (especially associated with underlying malignancy, neutropenia, and HIV human immunodeficiency virus, intravenous drug abuse, and indwelling catheters) [12]. Lung involvement by Aspergillus increases the risk of EE [13]. Due to the presence of anemia and lymphocytopenia in all patients, and renal failure in one patient, lowered immunity could have a role in the pathogenesis. Also, the history of abortion in one case may suggest the possibility of hematogenous spread of infection from a gynecological source.

The second possibility is direct viral infection. Retinal vasculitis, retinal degeneration and blood–retinal barrier breakdown had been confirmed in experimental animal models of coronavirus infection [14]. Positive PCR for conjunctival swabs was present in 2 patients who died from respiratory failure. Generally, the viral RNA quantities in conjunctival samples were much lower compared with those in respiratory specimens [6]. Angiotensin converting enzyme II (ACE 2) receptor is a cellular receptor for SARS-CoV-2 [15]. It has also been detected in the human retina [16], retinal pigment epithelium, choroid, and conjunctival epithelium [16–18]. In 2019, Smitha et al., reported a 10-year-old boy with viral orbital cellulitis secondary to H1N1 virus infection. The patient presented with pneumonia and fever and on the second day, he developed left ocular pain with swollen tense eyelids and mechanical ptosis. Magnetic resonance imaging (MRI) of orbits showed ethmoid and maxillary sinusitis and superior orbital cellulitis involving superior rectus muscle. The patient did not have proptosis and the diagnosis of orbital cellulitis relied only on MRI findings. Nasopharyngeal and throat swabs were taken for real-time polymerase chain reaction. Test was proven positive for H1N1 influenza virus. Following this, oseltamivir, a neuraminidase inhibitor, was given with significant reduction in temperature and complete resolution of the periorbital edema within 5 days [19]. In another case, bilateral viral endophthalmitis was reported as the presenting sign of severe immunodeficiency in an infant with presumed herpes simplex encephalitis, the condition simulated retinoblastoma with bilateral leukocoria at presentation [20]. Additionally, cytomegalovirus was reported to cause unilateral endophthalmitis with hypopyon and the opacity of vitreous body in an 83-year-old immune-competent female [21]. However, both viral endophthalmitis and orbital cellulitis at presentation is extremely rare with only few reported cases.

The third possibility is a multisystem inflammatory syndrome related to COVID-19. The risk of severe disease and mortality has been maximum in older patients and those with underlying noncommunicable diseases, such as hypertension, cardiac disease, chronic lung disease and cancer [22]. Recently, however, there are reports of cases of children and adolescents presented with multisystem inflammatory condition like Kawasaki disease and toxic shock syndrome with a presentation of acute illness accompanied by a hyperinflammatory syndrome, leading to multi-organ failure and shock [21–24]. Therefore, the unusual combination of endophthalmitis and orbital cellulitis could be explained as possible new presentation of the mysterious inflammatory syndrome linked to COVID-19.

The main limitation of our study is the small number of the patients but the combination of orbital cellulitis and endophthalmitis is a rare entity. However, we are presenting a new ophthalmic presentation of COVID-19. Further studies are needed to document similar cases and to explore the exact pathogenesis of this unusual presentation.

## Conclusion

Combined endophthalmitis and orbital cellulitis could be one of the early presentations in patients with COVID-19 infection with poor visual prognosis.

## Data Availability

All data generated or analysed during this study are included in this published article and are available from the corresponding author on reasonable request.

## Abbreviations

COVID-19: Coronavirus disease of 2019
MERS: Middle East respiratory syndrome
SARS: Severe acute respiratory syndrome
SARS-COV-2: Severe acute respiratory syndrome coronavirus-2
RNA: Ribonucleic acid
CT: Computed tomography
CBC: Complete blood count
PCR: Polymerase chain reaction
EE: Endogenous endophthalmitis
H1N1 virus: Swine flu virus
